# Natural history of shedding and household transmission of severe acute respiratory syndrome coronavirus 2 using intensive high-resolution sampling

**DOI:** 10.1371/journal.pone.0305300

**Published:** 2024-07-25

**Authors:** Jonathan Altamirano, Prasanthi Govindarajan, Andra L. Blomkalns, Sean Leary, India Robinson, Leanne X. Chun, Nuzhat J. Shaikh, Makeda L. Robinson, Marcela Lopez, Grace K-Y Tam, Yuan J. Carrington, Monique B. De Araujo, Katharine S. Walter, Jason R. Andrews, Julianne Burns, Catherine Hogan, Benjamin A. Pinsky, Yvonne Maldonado

**Affiliations:** 1 Department of Pediatrics, Stanford University School of Medicine, Stanford, California, United States of America; 2 Department of Epidemiology and Population Health, Stanford University School of Medicine, Stanford, California, United States of America; 3 Department of Emergency Medicine, Stanford University School of Medicine, Stanford, California, United States of America; 4 Department of Medicine-Division of Infectious Diseases, Stanford University School of Medicine, Stanford, California, United States of America; 5 Department of Epidemiology and Biostatistics, University of California, Irvine, California, United States of America; 6 Department of Pathology, Stanford University School of Medicine, Stanford, California, United States of America; The University of Hong Kong School of Public Health, HONG KONG

## Abstract

**Importance:**

The COVID-19 pandemic has led to 775 million documented cases and over 7 million deaths worldwide as of March 2024 and is an ongoing health crisis. To limit viral spread within households and in the community, public health officials have recommended self-isolation, self-quarantine of exposed household contacts, and mask use. Yet, risk of household transmission (HHT) may be underestimated due to low frequency of sampling, and risk factors for HHT are not well understood.

**Objectives:**

To estimate the secondary attack rate of SARS-CoV-2 within households and to define the risk factors for new infections in household members who are in close contact with the index case.

**Design, setting, and participants:**

In this prospective cohort study, from March 2020—December 2021 we enrolled 60 households with index cases who tested positive for SARS-CoV-2. All household contacts and index cases were tested daily for SARS-CoV-2 via reverse transcription polymerase chain reaction (RT-PCR) using self-collected anterior nares specimens. Households were followed until all study participants in the household tested negative for SARS-CoV-2 for seven consecutive days. We collected sex, age, race/ethnicity, comorbidities, and relationship to index case for secondary contacts, household level characteristics including primary income, household density, and square feet per person on property. We compared the sociodemographic variables between COVID-19 positive and negative household members and between households where secondary transmission did and did not occur.

**Main outcomes and measures:**

Daily anterior nares swabs were tested for SARS-CoV-2 using RT-PCR, in order to assess duration of nasal shedding of SARS-CoV-2, as well as risk of transmission to secondary household contacts.

**Results:**

Of the 163 participants in this study, 84 (51.5%) were women; median age (IQR) was 36.0 (17.0–54.0) years of age; 78 (47.8%) were white and 48 (29.5%) were Hispanic/LatinX. Of the fifty households with household contacts, at least one secondary case occurred in twenty-six households (52.0%) and forty-five household contacts (43.7%) were infected. **S**econdary attack rate was lowest among children of index cases (6/23, 26.1%). Modified Poisson regression identified that the risk of transmission to household contacts increases significantly with age (Risk ratio for each increase in years of age = 1.01, 95% CI = 1.00–1.02). Mixed effects regression models identified that participants with chronic diseases, such as asthma, diabetes, cancer, or cardiac disease, had higher Cts at baseline when compared to participants without chronic diseases (6.62, 95% CI: 1.46–11.77, p = 0.02) and show a slower rate of increase in Ct over time (-0.43, 95% CI: -0.77 to -0.09, p = 0.02)

**Conclusions and relevance:**

This study suggests that HHT represents a key source of community-based infection of SARS-CoV-2. Allocation of resources for contact investigations and prevention interventions should focus on the individuals at highest risk of infection in households, especially those with higher density homes.

## Introduction

The COVID-19 pandemic, caused by the novel severe acute respiratory syndrome coronavirus 2 (SARS-CoV-2), is a major global health crisis leading to over 775 million cases and 7 million deaths worldwide as of March 2024 [1]. This respiratory virus spreads from person to person via respiratory droplets and aerosols, as well as through contact with an infectious person or contaminated surface [2]. Given these modes of transmission, household contacts of an infected inhabitant are at high-risk for becoming infected with SARS-CoV-2. Meta-analyses from the early pandemic suggested that the household secondary attack rate (SAR) was around 17% (95% confidence interval [95% CI] = 14%– 21%), while later meta-analyses have estimates as high as 43% (95% CI = 35–50%), depending on variant [3, 4]. Demographic factors including age, gender, and socioeconomic status have all been associated with alterations in the SAR; however the effects of these parameters within households remains unclear [3–6]. In addition, while public health guidance for index cases within households has recommended self-isolation, mask wearing in shared spaces, and self-quarantine of exposed household contacts to reduce infection rates, the kinetics of SARS-CoV-2 transmission in these high-risk spaces are still poorly understood [7]. Here we describe a unique COVID-19 outpatient clinical cohort prospectively followed with daily SARS-CoV-2 testing to further define HHT rates within the California San Francisco Bay Area and better understand the factors associated with increased transmission risk between close contacts.

## Methods

### Study design and participants

Our study’s eligible population included all Stanford Health Care (SHC) outpatients with documentation of a SARS-CoV-2 positive nasopharyngeal swab between March 2020—December 2021. Potential participants were excluded if they were unable to provide consent, if they lived more than 50 miles away from Stanford and therefore would not be able to attend follow-up visits, or if their SARS-CoV-2 diagnostic samples were collected more than seven days before their potential enrollment date. Research staff contacted eligible patients to ascertain their willingness to participate and to obtain remote written consent from all eligible, consenting members of the household. Research staff identified the index case for each household based on earliest symptom onset of any confirmed cases, or else by earliest diagnosis if no symptoms were reported, and other household members identified as household contacts. During the enrollment visit, all participating household members completed structured questionnaires to collect the following data variables: 1) sociodemographic data such as age, sex, race, and occupation, 2) epidemiologic data, such as possible exposures to SARS-CoV-2 in the community, and 3) health data, such as symptom onset, symptom duration, date of SARS-CoV-2 diagnosis, and any chronic medical conditions to be used for subsequent analyses. All participants self-collected samples using anterior nares swabs for SARS-CoV-2 testing and completed daily symptom diaries for either 21 days from the date of study enrollment, or until every household member tested negative for seven consecutive days, depending on which happened first. If a participant was still testing positive after 21 days of follow-up, participants were given the option to stop sample collection, or else continue until all members of the household had tested negative.

### Sample testing, symptom duration, and shedding duration

Participants self-collected their first anterior nares swab (CLASSIQ Swabs; Copan Diagnostics) under the direct supervision of trained clinical staff who provided any corrections to technique as needed. After sample collection, samples were collected from households within four hours of self-collection and tested for SARS-CoV-2 using reverse transcription polymerase chain reaction (RT-PCR) targeting the envelope gene [8]. Cycle threshold (Ct) values were recorded for positive samples.

Symptom duration, for participants who reported symptoms, was defined as the time between the self-reported date of symptom onset to the self-reported end of symptoms during participants’ involvement in the study, as determined by their daily symptom diaries.

Shedding duration was measured differently for symptomatic and asymptomatic participants. For symptomatic participants, shedding duration was calculated as the time between either the date of symptom onset or date of first positive RT-PCR test, depending on which occurred first, to the date of their last positive RT-PCR test, confirmed after three consecutive negative tests. For asymptomatic participants, shedding duration was calculated as the time between the date of their first positive RT-PCR test to the date of their last positive RT-PCR test, confirmed after three consecutive negative tests.

### Statistical analysis

**Fig 1:**
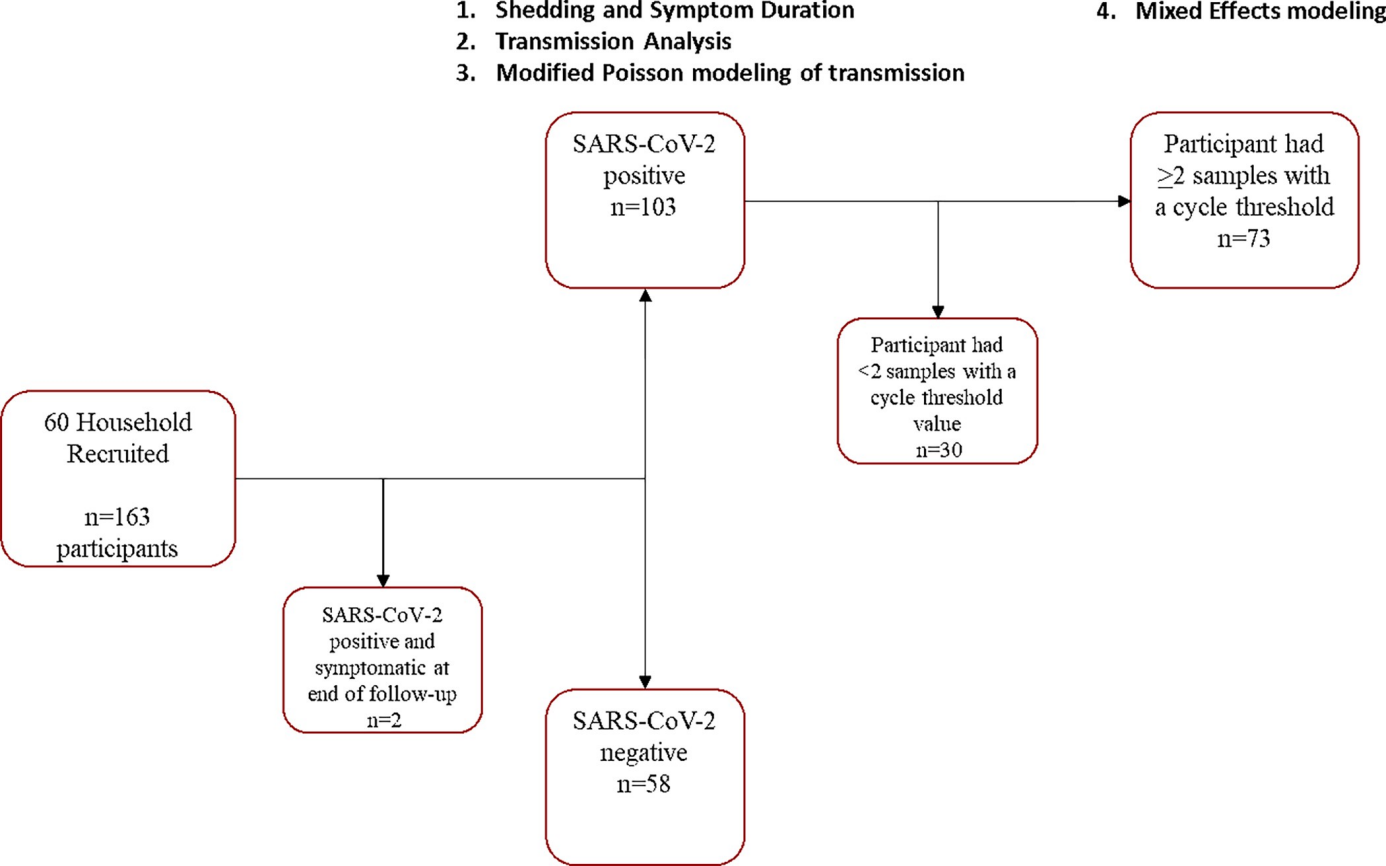
Enrollment study diagram. Enrollment diagram for the 163 participants recruited as part of this study. Diagram includes endpoints for participants, including positive and negative status, as well as which analyses were done with what population.

Here, we report participant and household demographic data, secondary attack rates (SAR) among households, and SAR among subgroups. Shedding and symptom duration by demographics were compared using the Kruskal-Wallis test. Number of household transmission events by demographics was compared between groups using Fisher’s Exact Test. A modified Poisson regression with robust standard errors was used to assess the association between risk of transmission to household contacts with age of household contact (in years) and chronic disease status of household contact (reported chronic disease vs no reported chronic disease). The Poisson model was adjusted for participant gender (men vs women), race (White, Asian, Black/African American, or Hispanic/LatinX), and by symptomatic status of index case (symptomatic vs asymptomatic. We evaluated change in Ct from Day 1—Day 20 using three mixed-effects linear regression models to analyze the association between Ct and: 1) symptom status (symptomatic vs asymptomatic household contacts), 2) chronic disease status (reported chronic disease vs no reported chronic disease), and 3) age (children under 18 vs adults 18 years and older). These mixed effect models included fixed effects (symptomatic status, chronic disease status, age, days since diagnosis, and the interactions between time and symptom status, chronic disease status, and age), and random effects (Ct measurements per individual). These models thus allow us to account for differences in Ct between groups at baseline as well as changes to Ct over time. As a parabolic relationship was suggested by graphing Ct over time after diagnosis, both time and time^2^ parameters were considered for each model. Final models were selected by using the Akaike information criterion for each model and selecting the model with the smallest criterion. Parameter estimates are presented here along with 95% confidence intervals (95% CI) and p-values. Please see Fig 1 for a visualization of participant follow-up across the study and for inclusion in the different analyses outlined here. Survey data were collected using REDCap surveys, a platform operated by the Stanford Medicine Research IT team. All data were summarized using SAS statistical software, version 9.4 (SAS Institute, Cary NC).

### Ethical review

The Stanford University’s Institutional Review Board approved (Protocol # 55479) this study. Remote consent was obtained from all participants. minimizing the exposure between research staff and participants.

## Results

### Participant characteristics

We enrolled 60 households with 163 participants. 60 participants were SARS-CoV-2 positive index cases and 103 were household contacts. Most participants were either non-Hispanic White (n = 78, 47.9%) or Hispanic/LatinX (n = 48, 29.5%). Women were slightly more likely to participate than men (n = 84, 51.5% vs. n = 79, 48.5%, respectively). The median age of participants was 36.0 years (Interquartile Range [IQR] = 17.0–54.0 years). Forty-two participants had been previously diagnosed with chronic medical conditions (25.8%), with the two most common conditions being asthma (n = 13, 31.0%) and diabetes (n = 8, 19.1%). Sociodemographic characteristics can be found in more detail in Table 1.

**Table 1:** Participant sociodemographic characteristics by infection status.

	Demographic Data				
Variable	Index Case	Household Contacts (HC)			Total
		Uninfected HC^a^	Infected HC^a^	Total HC	
**Participants, No.**	60	58 (56.3%)	45 (43.7%)	103	163
**Participant Gender, No. (%)**					
Men	28 (46.7)	29 (56.9)	22 (43.1)	51 (49.5)	79 (48.5)
Women	32 (53.3)	29 (55.8)	23 (44.2)	52 (50.5)	84 (51.5)
**Participant Age, Median, (IQR)**	40.5 (28.5–58.0)	31.0 (9.0–44.0)	40.0 (22.0–52.0)	34.0 (15.0–49.0)	36.0 (17.0–54.0)
**Participants by Age Group, No. (%)**					
Under 18	9 (15.0)	21 (65.6)	11 (34.4)	32 (31.1)	41 (25.1)
18–29	11 (18.3)	7 (77.8)	2 (22.2)	9 (8.7)	20 (12.3)
30–49	17 (28.4)	18 (47.4)	20 (52.6)	38 (36.9)	55 (33.7)
50–69	18 (30.0)	8 (50.0)	8 (50.0)	16 (15.5)	34 (20.9)
70+	5 (8.3)	4 (50.0)	4 (50.0)	8 (7.8)	13 (8.0)
**Participant Race/Ethnicity, No (%)**					
White	30 (50.0)	27 (56.3)	21 (43.7)	48 (46.6)	78 (47.8)
Asian	9 (15.0)	5 (35.7)	9 (64.3)	14 (13.6)	23 (14.1)
Black/African-American	2 (3.3)	6 (75.0)	2 (25.0)	8 (7.8)	10 (6.1)
Hispanic/LatinX	18 (30.0)	18 (60.0)	12 (40.0)	30 (29.1)	48 (29.5)
Declined to State	1 (1.7)	2 (66.7)	1 (33.3)	3 (2.9)	4 (2.5)
**Comorbidities, No. (%)**					
Any Chronic Health Condition (Ex: Asthma, Diabetes, Cancer)	16 (26.7)	12 (46.2)	14 (53.8)	26 (25.2)	42 (25.8)
**Relation to Index Case** ^**b**^					
Spouse/Partner	--	17 (53.1)	15 (46.9)	32 (31.1%)	--
Child	--	17 (73.9)	6 (26.1)	23 (22.3%)	--
Parent	--	11 (52.4)	10 (47.6)	21 (20.4%)	--
Sibling	--	8 (57.1)	6 (42.9)	14 (13.6%)	--
Child (Parent Caregiver)	--	0 (0)	4 (100)	4 (3.9%)	--
Grandchild	--	2 (66.7)	1 (33.3)	3 (2.9%)	--
Grandparent	--	1 (50.0)	1 (50.0)	2 (1.9%)	--
Child Caregiver (Nanny)	--	2 (100)	0 (0)	2 (1.9%)	--
Roommate	--	0 (0)	2 (100)	2 (1.9%)	--

a Row percentage reported for household contacts by infection status

b Reported only for household contacts

Of the 60 recruited households, ten (16.7%) were single person residences, while the other households had between two to seven household contacts, with a median of 3.0 people in a household (IQR = 2.0–4.0). These ten single-person households were excluded from any transmission analysis and only contributed shedding and symptom duration data.

### Household transmission

Of the 50 households with an index case and household contacts, transmission occurred in twenty-six households (n = 26/50, 52.0%), to forty-five household contacts (n = 45/103, 43.7%). In households with transmission, the SAR was lowest among the children of index cases, at 26.1% (n = 6/23) and highest among parents of pediatric index cases (n = 10/21, 47.6%) and spouses of index cases (n = 13/30, 43.3%). Based on self-reported symptom onset by participants, the median time between symptom onset for the index case and symptom onset in household contacts was three days (IQR = 2.0–9.0 days). Households with only one household contact were less likely to have a transmission event when compared to households with multiple contacts, although this was not statistically significant (43.5% vs 59.3% respectively, p = 0.27, χ^2^ = 1.23, 1 degree of freedom [df]). Of the sixteen households where multiple secondary contacts were at risk and a HHT event was noted, we observed secondary transmission to multiple household contacts in eleven households (68.8%). Households whose primary income came from science or medicine were less likely to have a HHT event (n = 10/23, 43.5%) when compared to other types of households, such as service and physical labor (n = 6/9, 66.7%, p = 0.43), although these differences were not statistically significant. More detail on household characteristics by transmission status can be found in Table 2.

**Table 2:** Household-level characteristics by transmission event.

Variable	Household Contacts Infection Status		Total
	No Transmission	Transmission	
**Household Characteristics, No. (%)**			
Households with any Household Contacts	24 (48.0)	26 (52.0)	50 (83.3)
Households with only one contact	13 (56.5)	10 (43.5)	23 (46.0)
Households with Multiple Contacts (>1)	11 (40.7)	16 (59.3)	27 (54.0)
Primary Income: Science or Medicine	13 (56.5)	10 (43.5)	23 (46.0)
Primary Income: Service or Physical Labor	3 (33.3)	6 (66.67)	9 (18.0)
Primary Income: Retired or Unemployed	3 (42.9)	4 (57.1)	7 (14.0)
Primary Income: Business or Finance	4 (57.1)	3 (42.9)	7 (14.0)
Primary Income: Government Employee	1 (33.3)	2 (66.7)	3 (6.0)
Refused to Response	0 (0)	1 (100)	1 (2.0)
**Number of Family Members, Median (IQR)**	2.0 (2.0–4.0)	3.0 (2.0–4.0)	3.0 (2.0–4.0)
**Square feet per person, Median (IQR)**	567.5 (400.0–809.3)	633.3 (503.5–875.8)	633.3 (400.0–875.8)

### Symptom and shedding duration

Median symptom duration, for symptomatic participants, was 18.5 days (IQR = 13.0–26.0 days), while shedding duration across participants was longer at 19.0 days (IQR = 13.0–31.0 days). Shedding and symptom duration demonstrated large heterogeneity, with symptom duration ranging from 3 to 63 days, and shedding duration from 6 to 62 days. Twenty-seven infected participants (25.7%) did not exhibit symptoms during their infection, including ten index cases and seventeen household contacts. Asymptomatic SARS-CoV-2 participants showed shorter shedding duration (13.0 days, IQR = 6.0–30.0 days) when compared to symptomatic participants (19.5 days, IQR = 15.0–31.0 days) (p = 0.03). Twenty-nine infected participants had chronic medical conditions. These participants showed similar shedding duration (18.0 days, IQR = 13.0–34.0) when compared to participants without chronic medical conditions (19.0 days, IQR = 13.0–30.0 days) (p = 0.77), and comparable symptom durations (17.0 days, IQR = 11.0–33.0 days vs 19 days, IQR = 13.0–26.0 days respectively) (p = 0.93). Shedding and symptom duration by sociodemographic factors can be found in Table 3. Shedding duration across demographics and stratified by symptomatic status can be found in S1 Table.

**Table 3:** Shedding and symptom duration for confirmed COVID-19 cases: Summary statistics.

Variable	Shedding Duration, Median (IQR)	p-value	Symptom Duration, Median (IQR)	p-value
**Participant Type**				
All Participants (n = 103)*	19.0 (13.0–31.0)		18.5 (13.0–26.0)	
Index Case (n = 58)	18.0 (13.0–33.0)	0.72	19.0 (10.0–26.0)	0.52
Household Contact (n = 45)	19.0 (13.0–28.0)		18.0 (14.0–26.0)	
**Symptom Status**				
Asymptomatic (n = 27) **	13.0 (6.0–30.0)	0.03	--	
Symptomatic (n = 76)	19.5 (15.0–31.0)		--	
**Participant Gender**				
Men (n = 48)	19.0 (13.0–30.5)	0.93	18.0 (14.0–26.0)	0.97
Women (n = 55)	19.0 (13.0–33.0)		19.0 (13.0–26.0)	
**Participants by Age Group**				
Under 18 (n = 20)	16.0 (10.5–30.5)	0.29	18.0 (12.0–26.0)	0.72
18–29 (n = 13)	26.0 (13.0–34.0)		23.5 (15.0–39.5)	
30–49 (n = 37)	19.0 (13.0–24.0)		15.0 (13.0–25.0)	
50–69 (n = 24)	18.0 (14.0–30.5)		21.0 (14.0–26.0)	
70+ (n = 9)	34.0 (18.0–39.0)		18.0 (9.0–39.0)	
**Patient Race/Ethnicity**				
White (n = 51)	19.0 (13.0–33.0)	0.73	18.0 (14.0–26.0)	0.39
Asian (n = 17)	19.0 (15.0–23.0)		21.5 (14.0–28.0)	
Black/African-American (n = 4)	18.0 (16.0–18.5)		14.5 (11.0–18.0)	
Hispanic/LatinX (n = 29)	20.0 (10.0–29.0)		21.0 (13.0–33.0)	
Declined to State (n = 2)	15.0 (13.0–17.0)		9.5 (7.0–12.0)	
**Comorbidities**				
No Comorbidities (n = 74)	19.0 (13.0–30.0)	0.77	19.0 (13.0–26.0)	0.93
Any Comorbidity (n = 29)	18.0 (13.0–34.0)		17.0 (11.0–33.0)	

* Two Index Cases excluded from shedding duration analysis. Participants asked to stop daily sampling after at least 21 days of follow up and remained positive. Participants were on days 27 and 29 post-symptom onset

** p <0.05

**Fig 2:**
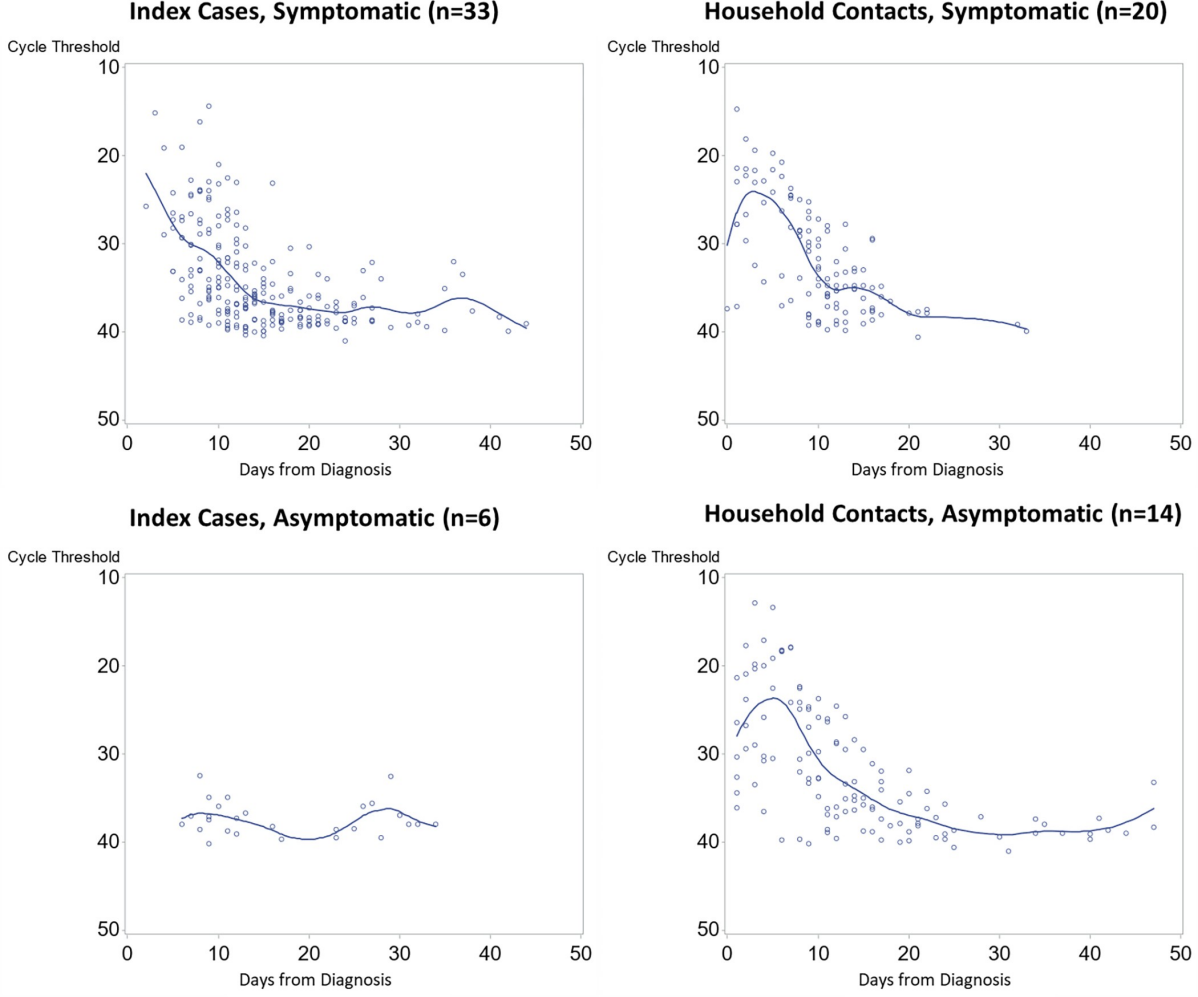
Cycle threshold over time for symptomatic and asymptomatic COVID cases. Cycle threshold over time since COVID-19 diagnosis by RT-PCR, stratified by index cases and household contacts as well as by symptomatic status. Each point represents a sample positive for SARS-CoV-2 collected from participants, with a local estimated scatterplot smoothing regression curve superimposed to provide a trend of cycle threshold over time.

Daily nasal swabs allowed us to quantify viral load over time for symptomatic and asymptomatic cases, as well as for index cases and household contacts, using RT-PCR cycle threshold Ct values visualized in Fig 2 (n = 503 samples). Viral load in household contacts increased over the first 6 days (average Ct change of -1.03/day), followed by a decrease in viral load (average Ct change of +0.72/day) that then plateaued at day 20. Viral load in symptomatic index cases increased steadily for the first 10 days (average Ct change of +0.83/day). Asymptomatic index cases showed little variation in their viral load (median Ct of 37.4, IQR = 33.8–38.8). Median Ct at diagnosis for index cases that did transmit SARS-CoV-2 to their household contacts were similar to median Ct at diagnosis for index cases that did not transmit to their contacts (respectively 27.0, IQR = 23.4–31.5 vs 26.8, IQR = 23.1–26.8, p = 0.68).

### Multivariate regression

The modified Poisson models identified a statistically significant one percent increase in risk of transmission to household contacts for each year increase in age of the contact (RR = 1.01, 95% CI = 1.00–1.02, p = 0.03). This model did not find a statistically significant increase in risk of transmission by chronic disease status of the household contact (RR = 1.38, 95% CI = 0.80–2.40, p = 0.25) (Table 4).

**Table 4:** Adjusted risk ratios (RR) and 95% confidence intervals (CI) for the association between age in years and chronic disease status with risk of transmission to household contacts.

	Crude RR (95% CI)	Adjusted RR (95% CI)	p-value
Age, years*	1.01 (1.00–1.02)	1.01 (1.00–1.02)	0.03
Any Chronic Disease	1.34 (0.85–2.09)	1.38 (0.80–2.40)	0.25

Poisson models with robust standard errors, adjusted for participant race, participant gender, and symptomatic status of index case. Chronic diseases include asthma, diabetes, cancer, hypertension, and chronic kidney disease.

*p<0.05

**Fig 3:**
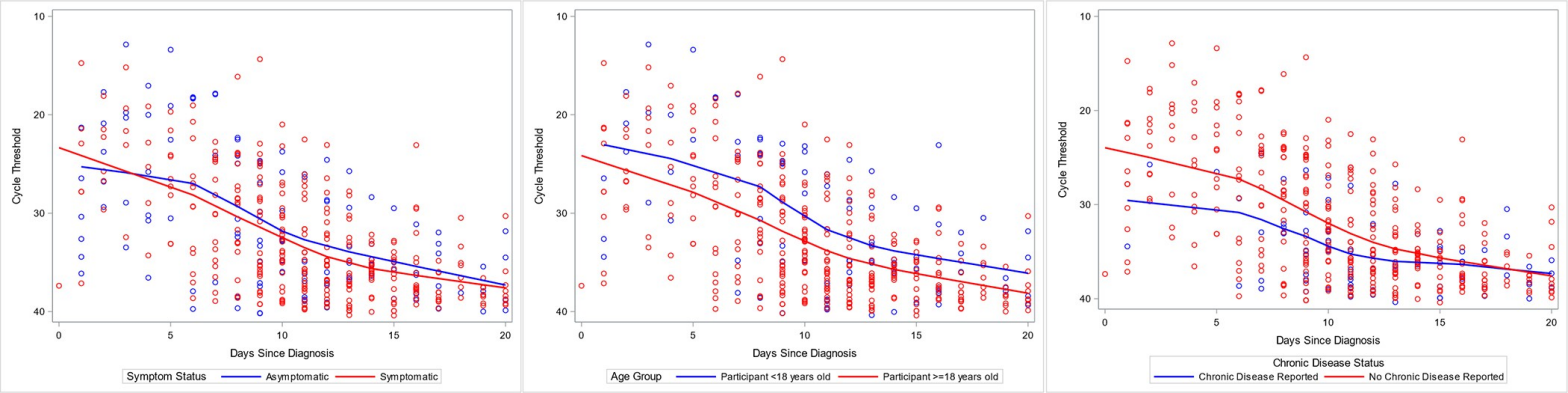
Cycle threshold over time by symptom status, age category, and chronic illness. Cycle threshold over time since COVID-19 diagnosis by RT-PCR, stratified by a) symptomatic status, b) age category, and c) reported chronic illness. Each point represents a sample positive for SARS-CoV-2 collected from participants, with a local estimated scatterplot smoothing regression curve superimposed to provide a trend of cycle threshold over time. Graph restricted to the first twenty days post-diagnosis.

Table 5 shows the results of the mixed-effects regression models. Participants with chronic diseases, such as asthma, diabetes, cancer, or cardiac disease, had higher Cts at baseline when compared to participants without chronic diseases (6.62, 95% CI: 1.46–11.77, p = 0.02) and show a slower rate of increase in Ct over time (-0.43, 95% CI: -0.77 to -0.09, p = 0.02). Children under 18 years old did not show significant differences in baseline Ct or in rate of change in Ct over time when compared to adults 18 years or older. Asymptomatic participants also did not show significant differences in baseline Ct or in rate of change in Ct over time. Changes in Ct over time by symptomatic status, age, and chronic disease status are visualized in Fig 3.

**Table 5:** Estimates and 95% CIs for cycle threshold by symptomatic status, age, and chronic disease status.

	Coefficient	95% CI	p-value
Symptomatic Status–Symptomatic vs Asymptomatic Infections			
Symptomatic	-0.22	-5.19 to 4.76	0.93
Days	0.83	0.59 to 1.07	0.0003
Symptomatic x Days	0.06	-0.29 to 0.42	0.73
Age–Children under 18 vs Adults 18 and Older			
Under 18	0.27	-6.02 to 6.56	0.93
Days	2.04	1.38 to 2.71	<0.0001
Under 18 x Days	-0.17	-0.57 to 0.23	0.42
Chronic Disease–Chronic Illness vs No Chronic Illness			
Chronic Disease	6.62	1.46 to 11.77	0.02
Days	0.82	0.66 to 0.97	<0.0001
Chronic Disease x Days	-0.43	-0.77 to -0.09	0.02

Results from three separate multilevel mixed-effect regression models including exposure of interest (symptomatic status, age, and chronic disease), time since either symptom onset or diagnosis, and the interaction between time and the exposures. Models include a random intercept and slope and group sample Ct by participants.

Chronic diseases include asthma, diabetes, cancer, hypertension, and chronic kidney disease.

## Discussion

This study of HHT includes intensive daily SARS-CoV-2 RT-PCR testing of all index cases and household members until shedding cessation to further define risk factors for HHT. Our study demonstrated a higher SAR within households (43.7%) than other estimates in literature, more than double the pooled mean from meta-analyses on the topic during the study period [3]. Our high-resolution sampling likely explains the higher SAR, as our daily testing likely allowed us to capture cases that would otherwise have been missed by less frequent sampling. Our data suggests that household-level factors may increase the risk for HHT, such as increased number of household members, as more transmission occurred within homes with multiple secondary contacts when compared to households with only one secondary contact, or type of occupation as a proxy for socioeconomic status, as households where income came primarily from service work saw greater risk of HHT when compared to occupations with higher salaries like science and medicine.

Our mixed-effects models identified differences in viral load trajectory for those with chronic diseases over the first 20-days post-diagnosis. As chronic illness has been well-documented to increase the risk of severe illness from COVID-19, elucidating the differences in viral load trajectory over time may help us hypothesize why these individuals are at such an increased risk of severe illness [9]. By contrast, our data did not reflect patterns seen in the literature, which suggest that asymptomatic cases of COVID-19 have different viral trajectories when compared to symptomatic cases [10]. However, this may simply be the result of small numbers. Our findings do align with estimates in the literature that asymptomatic cases make up over 20% of all COVID-19 cases, and that asymptomatic cases can still result in secondary infections [11].

As expected, those at greatest risk of transmission were members of the household who were anticipated to have prolonged contact with the index case, such as parents of index cases or adult caretakers of an elderly parent. These types of household contacts should be targeted in future outbreak control efforts, such as providing additional PPE and training to household contacts who need to continue caring for a sick family member. Of note, promising data regarding prophylactic monoclonal antibody studies have demonstrated reduced HHT on interim analysis and could be useful for control of HHT [12, 13]. Our data also suggest that age plays a role in transmission risk. The modified Poisson regression identified that risk of transmission to household contacts increases as the age of the contacts increases, even after adjusting for potential confounders like gender and race. Further, we saw that the SAR was lowest among children of index cases. These data add to the evidence that children are at a significantly lower risk of COVID-19 infection when compared to adults, important for public health officials to consider now that schools nationwide have reopened [14–17].

Shedding duration showed remarkable variation, between 6–62 days, and lasted longer on average than anticipated, given the five-day quarantine currently recommended by the CDC. While the CDC has shown that replication-competent virus is not detected after ten days of infection with SARS-CoV-2, our prolonged shedding duration does indicate that qualitative RT-PCR results are insufficient to make determinations regarding best isolation policies for patients. In the setting of asymptomatic infections, which are estimated to make up 20–30% of all COVID-19 cases, or pauci-symptomatic cases that do not prompt testing, someone well out of their 10-day window could be asked to quarantine unnecessarily after a positive COVID-19 test [18, 19]. More work will be needed in order to implement assays that better identify patients at risk for transmitting SARS-CoV-2.

Viral load decreases quickly over the course of infection, providing support for current CDC guidelines to limit the quarantine period to 5 days. In line with prior literature, our study identified that the first few days of infection have substantial heterogeneity but are generally marked by an increase in viral load, indicating potential increased risk in HHT during this early infectious period [20, 21]. We noted that household contacts had a faster decrease in viral load in our cohort when compared to index cases, although reasons for this difference are unclear. One possibility is that peak shedding among index cases may have occurred before their diagnosis, as index cases were recruited from outpatient care. Additionally, we found no difference in viral load between index cases that did and did not transmit the virus to their household contacts. This suggests that other factors beyond diagnostic viral load may play a crucial role in HHT, such as household density, the availability and use of intrahousehold masking and isolation to minimize transmission. In this way, continued education plays an essential role in minimizing HHT of COVID-19s, by providing households with a known case with key information to halt transmission.

Our study has some key limitations. First, we assume that transmission to household contacts is the result of secondary transmission from the household’s index COVID-19 case, based on quarantine protocols established by the CDC in the event of any COVID-19 case. However, without viral genome sequencing, we cannot confirm whether these contacts were infected via intrahousehold transmission or whether they received additional community exposure to SARS-CoV-2. Second, our participant households were not a representative sample of the San Francisco Bay Area, as certain populations, such as African-Americans and Asian-Americans, are underrepresented in our data. Finally, our analyses are exploratory and should be validated in other datasets. Finally, we must also note that transmission rates vary by strain and by vaccination rate, effects we cannot adjust for in this dataset, as strain data was not readily available throughout the study duration.

Our study also has multiple strengths. First, this is the first HHT study to prospectively follow index cases and all consenting household members using daily RT-PCR testing and symptom tracking that continued until all participants stopped shedding. This provides a highly accurate measure of shedding and symptom duration over the course of the study, in addition to the collection of extensive socio-demographic and clinical information. Second, we were able to recruit households with diverse socioeconomic backgrounds, with some in low-income service professions and others in higher income industries, such as computer science, allowing us to assess for potential HHT dynamics in distinct socioeconomic groups.

In summary, our data indicates that the rate of HHT of SARS-CoV-2 is high, in line with literature suggesting that roughly a quarter of infections are caused by household exposure and is likely a key contributor to community-based COVID-19 infection [22]. In addition to increasing SARS-CoV-2 vaccination rates and the possibility of administering prophylactic monoclonal therapy to eligible exposed household contacts, we anticipate that providing education, resources, and training to infected individuals may reduce the burden of HHT of SARS-CoV-2. Furthermore, it is indispensable to equip lower socioeconomic groups, who are at higher risk of COVID-19 infection and likely have higher secondary attack rates, with appropriate preventative resources to prevent HHT of SARS-CoV-2, contributing to a decrease in community spread and nationwide COVID-19 cases and deaths.

## Supporting information

S1 File (DOCX)

S1 Table (DOCX)
