# Supplementary material for: Natural history of shedding and household transmission of severe acute respiratory syndrome coronavirus 2 using intensive high-resolution sampling
Source: PLoS One. 2024 Jul 25;19(7):e0305300. doi: 10.1371/journal.pone.0305300 (PMC11271927; doi:10.1371/journal.pone.0305300)
Supplement: S1 File — (DOCX) [file pone.0305300.s001.docx]

**Supplemental Materials**

Details on remote consent and sample collection

Remote consent for participating household was collected over Zoom. Clinical research staff would walk through consent forms created in REDCap over a shared screen, to provide households with the opportunity to ask questions while minimizing contact with research staff. Participants were later emailed with a copy of their consent forms. In addition, our staff emailed a one-page instruction sheet with the steps to self-collect using anterior nares swabs and a short video animation demonstrating the sample collection method, in order to get participants acquainted with the sample collection technique. To ensure appropriate technique during sample collection, households were asked to collect specimens under the direct supervision of clinical staff on a video call. The samples were then picked up by the research staff at the participants’ homes. After collection, samples were placed in tubes filled with 3 mL of phosphate-buffered saline, stored in coolers, and were delivered to the Stanford Clinical Virology Laboratory within 4 hours of sample collection.

Details on REDCap platform

The REDCap platform services at Stanford are subsidized by a) Stanford School of Medicine Research Office, and b) the National Center for Research Resources and the National Center for Advancing Translational Sciences, National Institutes of Health, through grant UL1 TR001085.
