# Supplementary material for: Natural history of shedding and household transmission of severe acute respiratory syndrome coronavirus 2 using intensive high-resolution sampling
Source: PLoS One. 2024 Jul 25;19(7):e0305300. doi: 10.1371/journal.pone.0305300 (PMC11271927; doi:10.1371/journal.pone.0305300)
Supplement: S1 Table — (DOCX) [file pone.0305300.s002.docx]

| **Table S1: Shedding and Symptom Duration for Confirmed COVID-19 Cases: Summary Statistics** | | | | | |
| --- | --- | --- | --- | --- | --- |
|  |  | Symptomatic Cases (n=76) | |  | Asymptomatic Cases (n=27) |
|  |  | Shedding Duration in Days, Median (IQR) | Symptom Duration in Days, Median (IQR) |  | Shedding Duration in Days, Median (IQR) |
| **Participant Type** |  |  |  |  |  |
| Overall (n=103)* |  | 19.5 (15.0 – 31.0) | 18.5 (13.0 – 26.0) |  | 13.0 (6.0 – 30.0) |
| Index Case (n=58) |  | 18.5 (14.5 – 33.5) | 19.0 (10.0 – 26.0) |  | 14.5 (6.0 – 30.0) |
| Household Contact (n=45) |  | 21.0 (15.5 – 29.5) | 18.0 (14.0 – 26.0) |  | 13.0 (7.0 – 26.0) |
| **Participant Gender** |  |  |  |  |  |
| Men (n=48) |  | 20.5 (15.5 – 31.0) | 18.0 (14.0 – 26.0) |  | 15.5 (8.0 – 28.0) |
| Women (n=55) |  | 19.5 (14.5 – 32.0) | 19.0 (13.0 – 26.0) |  | 11.0 (6.0 – 34.0) |
| **Participants by Age Group** |  |  |  |  |  |
| Under 18 (n=20) |  | 17.0 (12.0 – 35.0) | 18.0 (12.0 – 26.0) |  | 13.0 (9.0 – 26.0) |
| 18 – 29 (n=13) |  | 27.5 (13.0 – 34.0) | 23.5 (15.0 – 39.5) |  | 13.0 (6.0 – 62.0) |
| 30 – 49 (n=37) |  | 19.0 (14.0 – 24.0) | 15.0 (13.0 – 25.0) |  | 13.0 (6.0 – 30.0) |
| 50 – 69 (n=24) |  | 20.0 (15.0 – 30.5) | 21.0 (14.0 – 26.0) |  | 14.5 (9.0 – 26.5) |
| 70+ (n=9) |  | 34.0 (18.0 – 39.0) | 18.0 (9.0 – 39.0) |  | -- |
| **Patient Race/Ethnicity** |  |  |  |  |  |
| White (n=51) |  | 19.5 (15.0 – 32.5) | 18.0 (14.0 – 26.0) |  | 13.0 (6.0 – 34.0) |
| Asian (n=17) |  | 19.5 (17.5 – 23.0) | 21.5 (14.0 – 28.0) |  | 11.0 (9.0 – 43.0) |
| Black/African-American (n=4) |  | 16.0 (14.0 – 18.0) | 14.5 (11.0 – 18.0) |  | 18.5 (18.0 – 19.0) |
| Hispanic/LatinX (n=29) |  | 22.0 (10.0 – 32.5) | 21.0 (13.0 – 33.0) |  | 11.0 (6.0 – 23.0) |
| Declined to State (n=2) |  | 15.0 (13.0 – 17.0) | 9.5 (7.0 – 12.0) |  | -- |
| **Comorbidities** |  |  |  |  |  |
| No Comorbidities (n=74) |  | 20.0 (15.0 – 30.5) | 19.0 (13.0 – 26.0) |  | 15.5 (7.0 – 30.0) |
| Any Comorbidity (n=29) |  | 18.5 (14.5 – 34.0) | 17.0 (11.0 – 33.0) |  | 11.0 (6.0 – 26.0) |
| * Two Index Cases excluded from shedding duration analysis. Participants asked to stop daily sampling after at least 21 days of follow up and remained positive. Participants were on days 27 and 29 post-symptom onset | | | | | |
